# Adverse reactions and efficacy of camrelizumab in patients with lung adenocarcinoma with high PD-L1 expression: A case report

**DOI:** 10.1097/MD.0000000000032731

**Published:** 2023-02-17

**Authors:** Tingting Wei, Zhisheng Wang, Xinlan Liu

**Affiliations:** a Department of Oncology, General Hospital of Ningxia Medical University, Yinchuan, Ningxia, P.R. China; b Laboratory Animal Center, Ningxia Medical University, Yinchuan, Ningxia, P.R. China.

**Keywords:** camrelizumab, case report, immunotherapy, irAEs, lung cancer

## Abstract

Immune checkpoint inhibitors have been rapidly developed for lung cancer therapy and major clinical guidelines have recommended them as the optimal first-line treatment for PD-L1-positive advanced lung cancer. Unfortunately, there is a lack of efficient prediction tools for the occurrence of immune-related adverse events (irAEs) caused by immunotherapy, and there is a lack of real-world data on the processing of irAEs, particularly those occurring in multiple systems simultaneously.

**Patient concerns:**

We report a 62-year-old male with expectoration who was diagnosed with lung adenocarcinoma with brain and bone metastases.

**Diagnoses:**

The results of the lung cancer tissue biopsy showed lung adenocarcinoma. Gene detection results of lung cancer tissue biopsy showed that the KRAS gene G12D was mutated and PD-L1 was positive, with a tumor proportion score of 95% (Dako 22C3 IHC platform).

**Interventions:**

The patient initially received 1 cycle of pemetrexed in combination with cisplatin-based chemotherapy. After the results of PD-L1 testing were reported, he received 1 cycle of camrelizumab immunotherapy in combination with pemetrexed plus cisplatin based chemotherapy.

**Outcomes:**

Seventeen days after treatment, the patient presented with symptoms such as yellow staining of the sclera and skin, itching throughout the body, dry mouth, and ecchymosis of the skin of the right lower extremity, which continued to worsen. Following treatment with 2 mg/kg methylprednisolone, the patient’s condition continued to deteriorate. IrAEs were controlled after dose escalation to 8 mg/kg in combination with plasma exchange therapy and treatment with multiple doses of mycophenolate ester. The patient then received no treatment for almost 2 months, but examination revealed that the tumor still had a persistent shrinkage reaction.

**Lessons:**

Camrelizumab has been well tolerated in several studies, but in patients with high PD-L1 expression and a G12D mutation in KRAS, one should be alert to the development of serious or even multisystem immune-related adverse effects. Timely and individualized selection of the hormone dosage is essential for the treatment of immunotherapy-induced multisystem irAEs.

## 1. Introduction

Lung cancer is the leading cause of morbidity and mortality worldwide and the curative effects of traditional therapies are limited. Programmed death receptor 1 (PD-1) and programmed death receptor ligand 1 (PD-L1) have made breakthroughs in the treatment of lung cancer in the last few years and are now the mainstay of treatment. This has opened up a new lung cancer treatment model and provided survival benefits for patients. Meanwhile, the curative effects of immunotherapy are accompanied by immune-related adverse events (irAEs). Severe irAEs will not only result in the interruption or termination of immunotherapy but will also put patients’ lives at risk. Effective prediction tools for irAE occurrence are currently lacking, and there is a lack of real-world data on the treatment of irAEs, particularly the simultaneous occurrence of multisystem. We used the PD-1 inhibitor, camrelizumab, in combination with chemotherapy to treat a patient with lung adenocarcinoma with high PD-L1 expression. The patient underwent a cycle of treatment with camrelizumab and subsequently developed multiple organ systems such as the liver, thyroid gland, pituitary gland, and myocardium. Remarkably, the patient did not receive antitumor therapy approximately 2 months later, but the tumor continued to shrink. The PD-L1 tumor proportion score (TPS) of the patient was 95%. Whether high expression of PD-L1 induces immune injury in multiple organs after 1 cycle of treatment or combined chemotherapy. To address this problem, we reviewed the related literature, discussed the relationship between TPS expression of PD-L1, clinical efficacy, and adverse events, and shared the treatment experiences of multiple organ irAEs.

## 2. Case report

### 2.1. Patient characteristics and previous treatment

**Figure 1.**
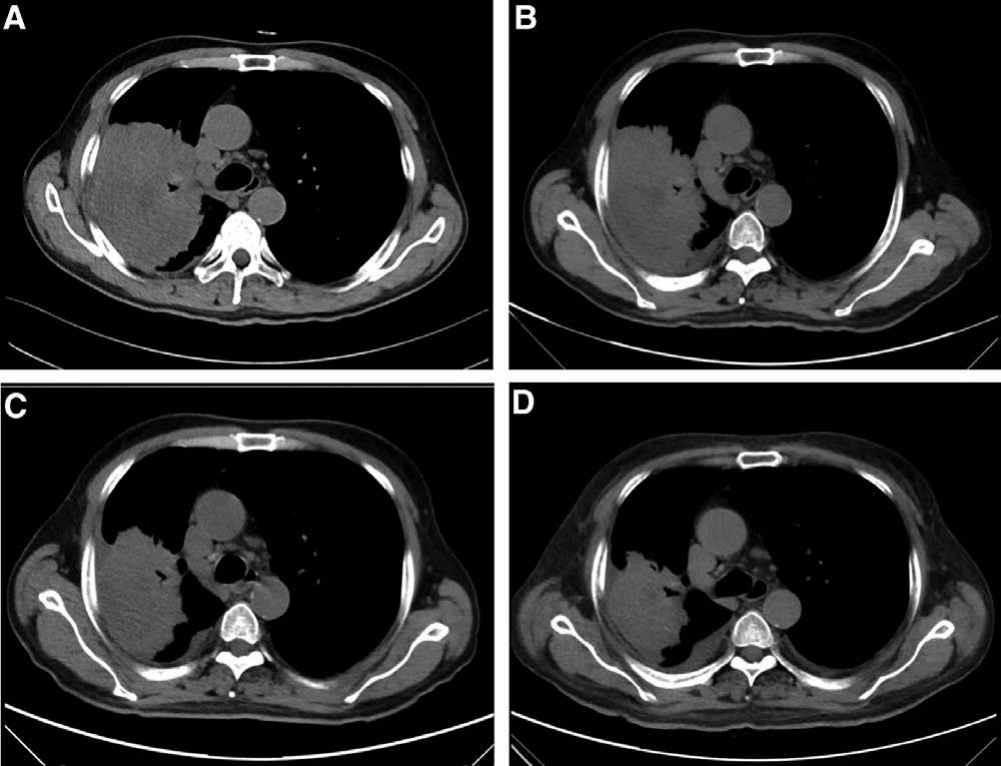
Chest CT images obtained following treatments (mediastinal window). At diagnosis (A), day 21 (B) after a cycle of chemotherapy with pemetrexed and carboplatin, the 28th day (C) and 59th day (D) after given a cycle of camrelizumab plus pemetrexed in combination with carboplatin. CT = computed tomography.

**Figure 2.**
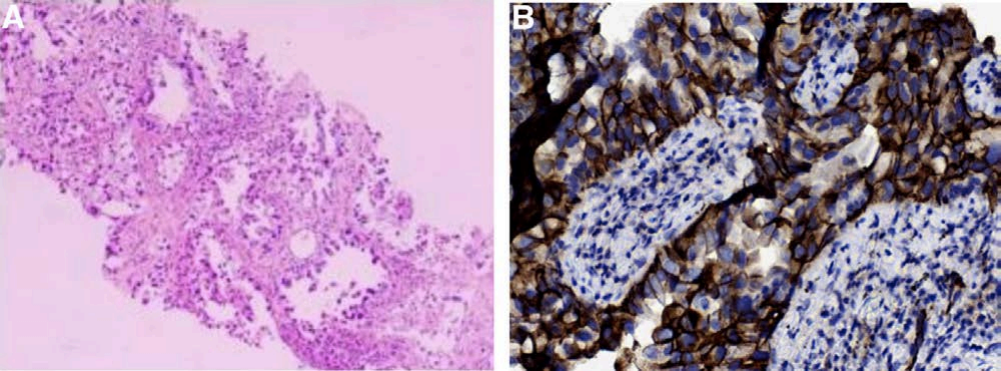
(A) Histological examination of the biopsy specimen of the right upper lobe of the lung showed adenocarcinoma; (B) immunohistochemical examination showed that the expression of PD-L1 (TPS) was 95.0%, with high expression, and the antibody type was 22C3. PD-L1 = programmed cell death-ligand 1. TPS = tumor proportion score.

**Figure 3.**
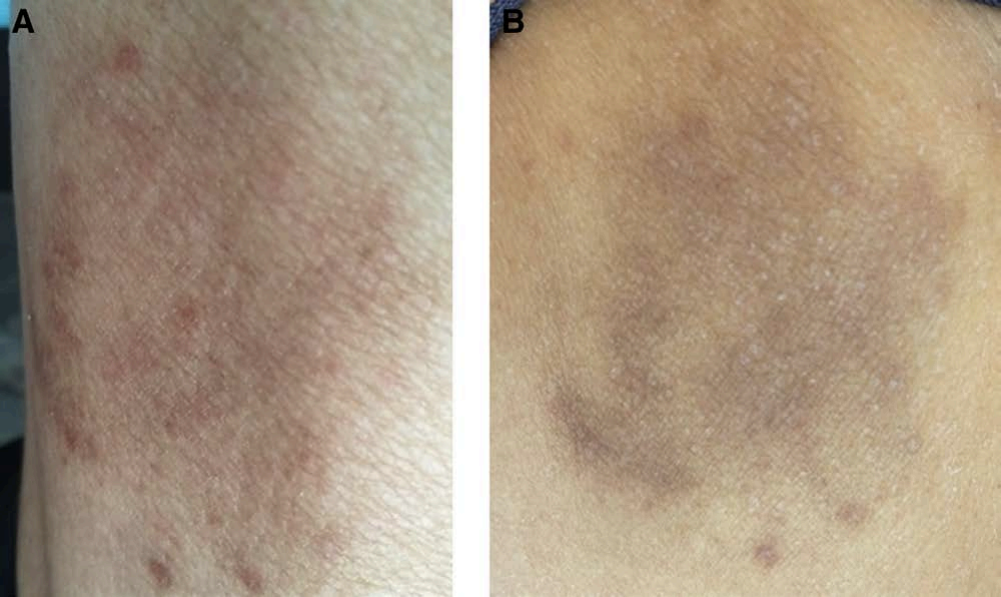
(A) Reactive cutaneous capillary hyperplasia (RCCEP) appeared on the right lower limb 17 days after the treatment with camrelizumab; (B) RCCEP subsided spontaneously after one week, with pigmentation. RCCEP = reactive cutaneous capillary hyperplasia.

The patient was a 62-year-old male weighing 59 kg. He was admitted to the hospital on December 21, 2021, because of “central adenocarcinoma of the right lung with brain and bone metastases for more than 2 months, then yellow staining of the skin and sclera for 7 days after 24 days of immunization combined with chemotherapy.” The patient presented to our hospital in early October 2021 for “coughing with blood in the sputum for over half a month.” Enhanced computed tomography scan showed that the pulmonary mass was located in the upper lobe of the right lung, which measured 11.0 × 8.7 cm. We considered cases of central lung cancer with obstructive pneumonia and multiple small mediastinum lymph nodes (Fig. 1A). Magnetic resonance imaging showed bilateral metastases to the frontal occipital lobe, and the patient did not have any obvious clinical symptoms of brain metastases. Bone scintigraphy revealed that local bone metabolism in the left acetabulum was active, and there was no evidence of bone destruction. On October 22, 2021, right lung adenocarcinoma was diagnosed via percutaneous lung biopsy (Fig. 2A) and a lung cancer tissue gene was detected. The TNM stage was cT4N1M1c stage IVB (8th TNM classification of lung cancer). On October 28, 2021, after 1 cycle of chemotherapy with pemetrexed 500 mg/m^2^ and carboplatin AUC 5, there was no change in the pulmonary lesions (11.0 × 8.2 cm) (Fig. 1B). On November 9, 2021, the results of gene detection in lung cancer tissues showed that the gene G12D of KRAS was mutated, and PD- L1 was positive (22C3 TPS was 95%) (Fig. 2B), while EGFR, ALK, MET, ROS1, RET, HER2, BRAF, NTRK, NRAS, and PIK3CA were all negative. On November 25, 2021, the regimen of “camrelizumab 200 mg on the first day, pemetrexed 500 mg/m^2^ on the second day, and carboplatin AUC 5 on the second day” was initiated. On December 12, 2021, the patient developed a yellow stain on his face and sclera, and skin ecchymosis on his right lower limb (Fig. 3A) [which subsided after 1 week (Fig. 3B)]. The symptoms gradually worsened and were accompanied by itching of the skin, dryness of the mouth, brown urine, white potting soil, general fatigue, and a poor appetite. The patient visited the hospital 1 week later and was considered to have an immune-related adverse event.

### 2.2. Diagnosis and treatment of irAEs caused by camrelizumab

Seventeen days after the use of camrelizumab, the patient developed irAEs with jaundice, dry mouth, rash, and fatigue as the primary manifestations, and his symptoms worsened. One week later, the hospital confirmed that the liver function was abnormal (TB > 10 × ULN, TB > 10 × ULN, ALT > 3 × ULN, AST > 5 × ULN, ALP > 20 × ULN), ACTH levels decreased, BNP levels increased, and T3 levels decreased. According to the evaluation standard of common adverse events of the National Cancer Institute (CTCAE 5.0), irAEs include immune hepatitis (G4 grade), reactive cutaneous capillary hyperplasia (G1 grade), low T3 syndrome, and adrenal insufficiency. Intravenous methylprednisolone (2 mg/kg) was administered according to the NCCN Management of Immunotherapy Related Toxicity 2021.^[1]^ After 3 days, liver function did not improve, and mycophenolate mofetil (1 g) was added twice a day. On the fifth day, none of the indices improved and the symptoms worsened. Methylprednisolone was added to 8 mg/kg, ceftriaxone sodium was administered to prevent infection, esomeprazole was administered to protect the gastric mucosa, vitamin D and calcium were supplemented to prevent osteoporosis induced by high-dose hormone therapy, and plasma exchange was provided for 3 consecutive days from the fifth day. Subsequently, liver function gradually improved, jaundice was relieved, dry mouth and fatigue symptoms were relieved, and urine color and stool color gradually returned to normal. Subsequently, the dosage of methylprednisolone was reduced every 5 days until the dosage was 32 mg for oral administration (Table 1).

**Table 1:** Laboratory examination and treatment medication of patients during treatment.

Time/day	TB (µmol/L)	DB (µmol/L)	ALT (U/L)	AST (U/L)	ALP (U/L)	ACTH (pg/mL)	NT-proBNP (pg/mL)	T3 (nmol/L)	PREDNIS (mg)	MMF (g)
D1	245.9	227.7	56.9	97	815	0.13	259.9	1.03	120	0
D3	254.6	217.3	98.0	148	645	0.19	827.3	0.72	120	1.0
D5	259.5	232.5	147.8	228	534	0.18	1747.0	0.89	480	1.0
D7	212.9	194.1	102.1	178	337	0.35	1247.0	0.82	480	1.0
D10	174.8	166.1	138.0	105	390	0.14	1202.0	0.78	480	1.0
D12	182.6	161.7	169.7	118	331	/	/	/	320	1.0
D15	174.5	158.6	194.3	92	347	0.10	970.8	0.48	320	1.0
D17	144.3	124.5	178.6	65	268	/	/	/	240	1.0
D20	112.2	110.6	189.0	73	314	/	/	/	240	1.0
D22	90.7	88.7	164.0	45	264	/	/	/	120	1.0
D24	85.0	76.5	130.0	45	244	/	/	/	120	1.0
D26	69.5	69.4	108.0	37	234	/	/	/	60	1.0
D31	63.6	61.9	71.6	26	201	/	/	0.7	32	1.0

Normal reference range of values: TB < 23 µmol/L, DB < 8 µmol/L, ALT < 41 U/L, AST < 40 U/L, ALP 40–129 U/L, ACTH 7.2–63.3 pg/mL, NT-proBNP < 125 pg/mL, T3 1.34–2.73 nmol/L.

ACTH = adrenocorticotropic hormone, ALP = alkaline phosphatase, ALT = alanine aminotransferase, AST = aspartate aminotransferase, DB = direct bilirubin, MMF = mycophenolate mofetil, NT-proBNP = the precursor of brain natriuretic peptide, PREDNIS = methylprednisolone, T3 = the serum triiodothyronine level, TB = total bilirubin.

### 2.3. Changes of patients’ pulmonary mass during treatment

An adenocarcinoma of the right upper lobe of the patient’s lung was found to be 11.0 × 8.7 cm in size (Fig. 1A). After 1 cycle of pemetrexed plus carboplatin chemotherapy before the gene test results were reported, the pulmonary mass size was 11.0 × 8.2 cm (Fig. 1B), and the disease was stable after curative effect evaluation (Evaluation Standard for Solid Tumor Efficacy Response Evaluation Criteria in Solid Tumors Version 1.1). After gene detection, given 1 cycle of camrelizumab and pemetrexed combined with carboplatin, the mass was reduced to 9.5 × 6.2 cm (Fig. 1C) on the 28th day and 8.7 × 4.5 cm (Fig. 1D) on the 59th day after treatment. Evaluation of effectiveness: partial response (PR) of the disease.

## 3. Discussion

In recent years, various clinical trials of immunotherapy in lung cancer have been carried out continuously, such as CheckMate-017, 057,^[2]^ KEYNOTE-010,^[3]^ and OAK,^[4]^ which have confirmed the significant overall survival benefit of immunotherapy. Camrelizumab (SHR-1210) is a potent monoclonal anti-PD-1 antibody from the China Hengrui Pharmaceutical Company and is used in the treatment of cancer. Camel study ^[5]^ showed that the median OS of patients with advanced non-small cell lung cancer (NSCLC) was 27.9 months after chemotherapy and the ORR was 60.5%. The Chinese Society of Oncology (CSCO) NSCLC Guidelines (2021) ^[6]^ recommend camrelizumab in combination with pemetrexed and platinum as a first-line treatment plan for advanced NSCLC with negative driver gene mutations (Class 1A evidence recommended at Level II), and camrelizumab has been included in the National Medical Insurance Catalogue. In the Camel study, the most common irAEs in the camrelizumab combined with chemotherapy group included reactive cutaneous capillary hyperplasia (78%, ≥ grade 3 < 1%), elevated alanine aminotransferase (12%), elevated aspartate aminotransferase (11%), related hepatitis (11%), and hypothyroidism (10%), most of which were grade 1 or 2.^[5]^ Two real-world studies have shown that the adverse reactions of camrelizumab has good efficacy and tolerance in patients with advanced lung cancer in China.^[7,8]^ The patient developed grade 4 immune hepatitis and grade 1 reactive cutaneous capillary hyperplasia after 1 cycle of camrelizumab treatment. During treatment, there was low T3 syndrome, decreased ACTH, and increased BNP, especially immune hepatitis.

The incidence of immune checkpoint inhibitors (ICI)-related hepatitis is 5% to 30%,^[9,10]^ which is mainly manifested by an increase in alanine aminotransferase (ALT) and/or aspartate aminotransferase (AST) with or without bilirubin. No adverse reactions to grade 4 bilirubin increase have been reported in related studies of camrelizumab. Many reports indicate that the median attack time for immune hepatitis is approximately 8 to 12 weeks after ICIs.^[1]^ Studies have shown that^[11,12]^ expression level of PD-L1 is positively correlated with efficacy, and pembrolizumab in advanced NSCLC patients with a TPS ratio ≥50% is significantly higher than that in patients with low expression in tumor reaction, progression free survival, and overall survival; however, the relationship between adverse events and TPS ratio is not mentioned. Compared to patients without irAEs, patients with grade 1 to 2 irAEs in immunotherapy have longer OS, whereas patients with grade ≥ 3 irAEs have the shortest OS, which is considered to be caused by treatment interruption or termination.^[13]^ Currently, there are no accurate predictive factors or predictive models for the risk of irAEs.^[14]^ Jing Y^[15]^ reported that PD-1 and PD-L1 expression is correlated with the occurrence of irAEs. In this case, TPS expression of PD-L1 was as high as 95%. After 17 days of treatment with camrelizumab, various irAEs appeared, including grade 4 immune hepatitis with elevated bilirubin levels as the main manifestation. The treatment was stopped immediately, but the tumor continued to shrink 59 days after treatment was stopped. It may be considered that the higher the TPS ratio of PD-L1 expression, the earlier the occurrence of irAEs, the more severe it is, and the more significant the tumor reaction. However, large-scale clinical trials are required to confirm these findings.

Studies have shown that patients who have already suffered from pulmonary inflammatory reactions, dermatitis, and hypothyroidism are most likely to develop multisystem irAEs.^[16]^ While PD- L1 inhibitor combined with chemotherapy can reduce the overall incidence of irAEs.^[17]^ Patients with advanced non-small cell lung cancer were treated with PD-L1 ICIs, and multisystem irAEs were associated with a better curative effect of immunotherapy.^[18]^ In this case, the patient had no previous treatment history with other PD-L1 ICIs and the first simple chemotherapy was well tolerated. Multisystem irAEs appeared after 1 cycle of camrelizumab combined with chemotherapy, but the tumor continued to shrink after the treatment was terminated, which is consistent with the report.

Currently, hormone therapy is the primary treatment for irAEs. The NCCN guidelines suggest that ICIs should be permanently stopped for grade 4 immune hepatitis, and the recommended dosage of methylprednisolone is 1 to 2 mg/kg. After 3 days, the liver function did not improve; therefore, mycophenolate mofetil (500–1000 mg Q12H) was added.^[1]^ Yue et al pointed out that the addition of hormones to grade 4 immune hepatitis is beneficial for better control of the autoimmune inflammatory reaction; however, the risk of opportunistic infections also increases.^[19]^ The patient’s liver function deteriorated further after treatment with methylprednisolone (2 mg/kg/day) for 5 days. The patient’s condition was controlled by adjusting the methylprednisolone dose to 8 mg/kg/day condition was controlled. Close attention should be paid to the various adverse reactions caused by high-dose hormones, and preventive anti-infection and acid suppression measures should be implemented to protect the gastric mucosa. Analysis of the treatment of this patient, after considering the treatment of irAEs, the dosage of hormone can be individually increased, and at the same time, immunosuppressant, immunomodulator and plasma exchange therapy can be appropriately combined.

The incidence of KRAS G12D mutation in NSCLC is approximately 4%, accounting for 15% of all KRAS mutations.^[20,21]^ Patients with G12D mutations had significantly lower median PD-L1 TPS expression than those with non-G12D mutations (1% vs 5%, *P* = .002). Patients with G12D mutations receive ICIs therapy with a lower objective response rate and worse prognosis than those with non-G12D mutations and immune combination, which may be a better treatment modality for patients with G12D mutations.^[22]^ The patient with the KRAS G12D mutation in this case, but with high expression of PD-L1 (TPS = 95%), was administered 1 cycle of chemotherapy combined with immunotherapy. However, whether this caused multisystem immune-related adverse effects associated with the KRAS G12D mutation remains to be further explored.

In summary, by analyzing the diagnosis and treatment of irAEs caused by lung cancer treated with camrelizumab in this case, we know that irAEs may occur at any time after ICIs are first used, and this requires close monitoring. The TPS ratio of PD-L1 expression may be an effective index for predicting the time of occurrence, severity, and tumor reaction to irAEs. Multisystem irAEs are associated with the improved curative effects of immunotherapy. Hormones are basic medications used to treat irAEs. Individualized and timely adjustment of hormone dosage and other adjuvant therapies are keys to the success of irAE treatment.

## Author contributions

**Conceptualization:** Tingting Wei, Zhisheng Wang, Xinlan Liu.

**Formal analysis:** Zhisheng Wang.

**Writing – original draft:** Tingting Wei.

**Writing – review & editing:** Xinlan Liu.
